# Sero-Prevalence and Sero-Incidence of Antibodies to SARS-CoV-2 in Health Care Workers in Israel, Prior to Mass COVID-19 Vaccination

**DOI:** 10.3389/fmed.2021.689994

**Published:** 2021-06-24

**Authors:** Khitam Muhsen, Mitchell J. Schwaber, Jihad Bishara, Eias Kassem, Alaa Atamna, Wasef Na'amnih, Sophy Goren, Anya Bialik, Jameel Mohsen, Yona Zaide, Nimrod Hazan, Ortal Ariel-Cohen, Regev Cohen, Pnina Shitrit, Dror Marchaim, Shmuel Benenson, Debby Ben-David, Bina Rubinovitch, Tamar Gotessman, Amir Nutman, Yonit Wiener-Well, Yasmin Maor, Yehuda Carmeli, Dani Cohen

**Affiliations:** ^1^Department of Epidemiology and Preventive Medicine, School of Public Health, Sackler Faculty of Medicine, Tel Aviv University, Tel Aviv, Israel; ^2^National Institute for Antibiotic Resistance and Infection Control, Israel Ministry of Health, Tel Aviv, Israel; ^3^Sackler Faculty of Medicine, Tel Aviv University, Tel Aviv, Israel; ^4^Infectious Diseases Unit, Rabin Medical Centre, Beilinson Hospital, Petah-Tiqva, Israel; ^5^Department of Pediatrics, Hillel Yaffe Medical Centre, Hadera, Israel; ^6^Department of Cardiology, Hillel Yaffe Medical Centre, Hadera, Israel; ^7^American Medical Laboratories, Herzlya, Israel; ^8^Infection Control Unit, Sanz Medical Centre, Netanya, Israel; ^9^Infection Control Unit, Meir Medical Centre, Kefar Saba, Israel; ^10^Infection Control Unit, Shamir (Assaf Harofeh) Medical Centre, Be'er Ya'akov, Israel; ^11^Department of Clinical Microbiology and Infectious Diseases, Hadassah Hebrew University Medical Centre, Jerusalem, Israel; ^12^Infection Control Unit, Wolfson Medical Centre, Wolfson, Israel; ^13^Infection Control Unit, Beilinson Hospital, Rabin Medical Centre, Petah-Tiqva, Israel; ^14^Infectious Disease and Infection Control Service, Hasharon Hospital, Rabin Medical Centre, Petah-Tiqva, Israel; ^15^Infectious Disease Unit, Shaare Zedek Medical Centre, Jerusalem, Israel; ^16^Infectious Disease Unit, Wolfson Medical Centre, Wolfson, Israel

**Keywords:** health care workers, sero-epidemiology, SARS-CoV-2, nucleocapsid antigen, risk factors, longitudinal study, occupational risk

## Abstract

**Objectives:** This study aims to examine the prevalence and risk factors of severe acute respiratory syndrome coronavirus 2 (SARS-CoV-2) sero-positivity in health care workers (HCWs), a main risk group, and assess the sero-incidence of SARS-CoV-2 infection between the first and second waves of coronavirus disease 2019 (COVID-19) in Israel.

**Methods:** A longitudinal study was conducted among 874 HCWs from nine hospitals. Demographics, health information, and blood samples were obtained at baseline (first wave—April–May 2020) and at follow-up (*n* = 373) (second wave—September–November 2020). Sero-positivity was determined based on the detection of total antibodies to the nucleocapsid antigen of SARS-CoV-2, using electro-chemiluminescence immunoassay (Elecsys® Anti-SARS-CoV-2, Roche Diagnostics, Rotkreuz, Switzerland).

**Results:** The sero-prevalence of SARS-CoV-2 antibodies was 1.1% [95% confidence intervals (CI) 0.6–2.1] at baseline and 8.3% (95% CI 5.9–11.6) at follow-up. The sero-conversion of SARS-CoV-2 serum antibody was 6.9% (95% CI 4.7–9.9) during the study period. The increase in SARS-CoV-2 sero-prevalence paralleled the rise in PCR-confirmed SARS-CoV-2 infections among the HCWs across the country. The likelihood of SARS-CoV-2 sero-prevalence was higher in males vs. females [odds ratio (OR) 2.52 (95% CI 1.05–6.06)] and in nurses vs. physicians [OR 4.26 (95% CI 1.08–16.77)] and was associated with being quarantined due to exposure to COVID-19 patients [OR 3.54 (95% CI 1.58–7.89)] and having a positive PCR result [OR 109.5 (95% CI 23.88–502.12)].

**Conclusions:** A significant increase in the risk of SARS-CoV-2 infection was found among HCWs between the first and second waves of COVID-19 in Israel. Nonetheless, the sero-prevalence of SARS-CoV-2 antibodies remains low, similar to the general population. Our findings reinforce the rigorous infection control policy, including quarantine, and utilization of personal protective equipment that should be continued together with COVID-19 immunization in HCWs and the general population.

## Introduction

The coronavirus disease 2019 (COVID-19) pandemic, caused by severe acute respiratory syndrome coronavirus 2 (SARS-CoV-2) (1), poses a huge health and societal burden globally. SARS-CoV-2 is easily transmitted from person to person (2) via droplets from the respiratory tract of infected people, including asymptomatic individuals (2, 3). COVID-19 may be severe and result in death, particularly among the elderly and persons with chronic diseases (4–6).

Health care workers (HCWs) comprise a main occupational risk group for SARS-CoV-2 infection (6, 7). Accordingly, special attention should be given to this group to maintain functional healthcare systems, including preventive measures, ensuring the availability of personal protective equipment, assessment of exposure, and prioritization in vaccination with COVID-19 vaccines. A survey conducted toward the end the first COVID-19 wave (April–May 2020) in Israel showed a low prevalence (0.2%) of asymptomatic SARS-CoV-2 infection among HCWs (8) as confirmed by PCR. A second wave of COVID-19 occurred in Israel with a peak in mid-September 2020; this resulted in a second lockdown during September–October 2020. A third wave of COVID-19 during December 2020–January 2021 resulted in a third lockdown, coinciding with a mass vaccination campaign targeting the adult population and HCWs, using the BNT162b2 mRNA COVID-19 vaccine (9, 10).

Sero-epidemiological studies can provide a more sensitive tool for the assessment of exposure to SARS-CoV-2 than molecular assays that identify only current infection. Sero-epidemiological studies in HCWs have shown variable sero-positivity for SARS-CoV-2 serum antibodies, with low estimates between 1.3 and 4.0% in Greece, Germany, and Denmark (11–13) and higher estimates ranging from 10 to 31.6% in the United Kingdom, Spain, Sweden, and some regions in the United States (14–16). Within-country variation in SARS-CoV-2 antibody sero-prevalence among HCWs was also reported (16). For example, a multicenter study in the United States showed point estimates ranging from 0.8 to 31.6% (16), with generally higher sero-prevalence found in HCWs from communities with higher incidence rates of COVID-19 (16). Most studies on the prevalence of SARS-CoV-2 antibodies in HCWs were cross-sectional and typically captured the initial months of COVID-19 surge (11, 13). Moreover, evidence on the risk factors for SARS-CoV-2 sero-prevalence among HCWs remains elusive and conflicting (11, 12, 15). For example, some studies showed higher SARS-CoV-2 antibody sero-positivity in HCWs of hospitals or departments designated for the treatment of COVID-19 patients compared to HCWs who worked in non-COVID-19 hospitals/departments (12, 13). Other studies, however, found no significant differences in SARS-CoV-2 serum antibodies between HCWs of COVID-19 units, or those who were involved in COVID-19 treatment, than those who were not (11, 15). Another conflicting issue is whether the risk of SARS-CoV-2 differs according to profession of HCWs (e.g., nurses, physicians, and technicians) (15–17). Such evidence is highly important for the enhancement of preventive measures to mitigate COVID-19 risk among HCWs. Accordingly, the aim of the current study was to examine the prevalence of and risk factors for SARS-CoV-2 sero-positivity in HCWs and to assess the sero-incidence between the first and second waves of COVID-19 in Israel. We also described the incidence of PCR-confirmed SARS-CoV-2 infection in HCWs in Israel.

## Materials and Methods

### Study Design and Population

Baseline sero-epidemiological studies were undertaken during April–May 2020 (the end of the first wave of COVID-19 in Israel) among HCWs [physicians, nurses, and others (technicians and administrative staff)], employees of nine general medical centers in Israel: Shamir (Assaf Harofe), Beilinson, HaSharon, Meir, Wolfson, Hadassah Ein Kerem, Sha'arei Zedek, Laniado, and Hillel Yaffe medical centers. Demographic and health information were collected using self-administered questionnaire, and a blood sample was obtained. A follow-up assessment was performed in a sub-sample of the participants, employees of five out of the nine medical centers, during September–November 2020 (the second COVID-19 wave).

Publicly available aggregated data on the number of HCWs, employees of hospitals, who had PCR-confirmed SARS-CoV-2 infection was obtained. This information is reported to the Ministry of Health by the general hospitals, public and private, in Israel.

### Study Variables

The main dependent variables are the prevalence of serum antibodies against SARS-CoV-2 at baseline and at the follow-up assessments, which provides a picture of change over time and cumulative infection burden. Sero-conversion (sero-incidence) was defined as positive serological results at the follow-up assessment among HCWs who tested negative at the baseline assessment, which measures the rate at which new infections occurred. Laboratory-confirmed COVID-19 was defined as a positive PCR test result for SARS-CoV-2 RNA, based on the participant's report.

The main independent variables were defined based on self-reports of the participants in the study questionnaire. The independent variables included the following: (i) demographics (age and sex); (ii) occupational characteristics {profession [physician, nurse, or other (e.g., administrative staff and nursing assistants)], years working in the profession, and working in a coronavirus department}; and (iii) types of COVID-19-related exposures, which were defined based on multiple questions. Participants were asked whether they were exposed to COVID-19 patients in the past 3 months and whether they were requested to be in quarantine due to exposure to COVID-19 patients in the past 3 months. The rationale to ask about these two levels of exposure was that HCWs were given an exemption from quarantine if the exposure to a COVID-19 patient occurred while adequately using personal protective equipment (i.e., while being at work in the hospital). Exposures of HCWs to COVID-19 patients might occur outside work, while being at work, but not adequately using personal protective equipment, or unprotected exposure to infected co-workers (e.g., during breaks); under these circumstances, HCWs were asked to be in quarantine. Information was also obtained on quarantine of family members due to exposure to COVID-19 patient in the past 3 months. Participants were also asked whether they performed PCR test for the detection SARS-CoV-2 and for the results of the test.

Information was also obtained on clinical symptoms of COVID-19 among HCWs.

### Laboratory Methods

Blood specimens were collected and transferred in cooled conditions immediately after collection to the study laboratory at Tel Aviv University. The samples were centrifuged and aliquots of the serum were frozen at −80°C until testing. At baseline assessment, the levels of serum immunoglobulin G (IgG) antibody against the spike (S) protein of SARS-CoV-2 were measured by an enzyme-linked immunosorbent assay (ELISA), using a validated commercial kit (EUROIMMUN AG, Luebeck, Germany), according to the manufacturer's instructions. The reported sensitivity of this assay ranged between 93.8 and 100%, 2–3 weeks after symptom onset, and its specificity ranged between 95.6 and 99.3% (18–20). All specimens with positive and borderline results and a randomly selected subset with negative results (total 69 samples) were retested using an electrochemiluminescence immunoassay (ECLIA) (Elecsys® Anti-SARS-CoV-2, Roche Diagnostics, Rotkreuz, Switzerland) for the detection of total antibodies (including IgG) to SARS-CoV-2 nucleocapsid (N) antigen. The tests were run on the Cobas 6000 e601 analyzer in collaboration with American Medical Laboratories, Herzliya, Israel. The sensitivity and specificity of the assay were reported at 89 and 100% (21), respectively. Sero-positive participants at baseline were classified based on positive results in both the Roche and EUROIMMUN kits; otherwise, participants were classified as sero-negative. This strategy was used to lower the potential of false positive results. At follow-up, only the Elecsys® Roche kit was used, and samples were classified as positive or negative using this kit. The laboratory tests were performed in a blinded manner to the background characteristics of the participants.

### Statistical Methods

Characteristics of participants were described using means and standard deviations (SD) for continuous variables and counts and percentages for categorical variables. Both at baseline and follow-up, the proportion [and 95% confidence intervals (CI)] of participants with SARS-CoV-2 serum antibodies (sero-prevalence) was calculated as the number of participants with positive results out of all tested participants. The sero-incidence rate was calculated as the proportion of participants who tested positive for SARS-CoV-2 serum antibodies, among participants who tested sero-negative at baseline.

Differences between participants with and without SARS-CoV-2 serum antibodies in demographic and occupational characteristics and possible exposures to the SARS-CoV-2 were examined using the chi-square test for categorical variables and Student's *t*-test for continuous variables. For each independent variable, odds ratio (OR) and 95% CI were calculated using logistic regression models. Adjusted associations were obtained by multivariable logistic regression models. The selection of independent variables to be included in the multivariable models was based on prior evidence of possible association between the variable and the risk of SARS-CoV-2 infection and our hypotheses of possible association between variables of interest and SARS-CoV-2 antibody sero-positivity (e.g., sex, profession, and being in quarantine due to exposure to a confirmed COVID-19 patient). Variables associated with SARS-CoV-2 sero-prevalence with *P* < 0.2 in the bivariate analysis were assessed in the multivariable models. In case of highly correlated variables, only one was included in the model. For example, since the variables “ever worked in a coronavirus department” and “working in a coronavirus department in the last 3 months” were highly correlated (phi correlation coefficient 0.82, *P* < 0.001), we assessed only one of these variables in the multivariable models. Since our sample of sero-positive individuals was modest, our aim was to include four–five variables in the multivariable model (22). Since it is expected that HCWs who had a positive PCR test results will be most likely sero-positive for SARS-CoV-2 antibodies, we conducted two models, one with and one without the variable “positive SARS-Cov-2 PCR test results.” This approach was followed to enable the identification of risk factors for SARS-CoV-2 transmission among HWCs.

*P* < 0.05 was considered statistically significant. Data were analyzed using SPSS version 27 (Armonk, NY: IBM Corp).

## Results

### Incidence of SARS-CoV-2 Infection in HCWs

**Figure 1:**
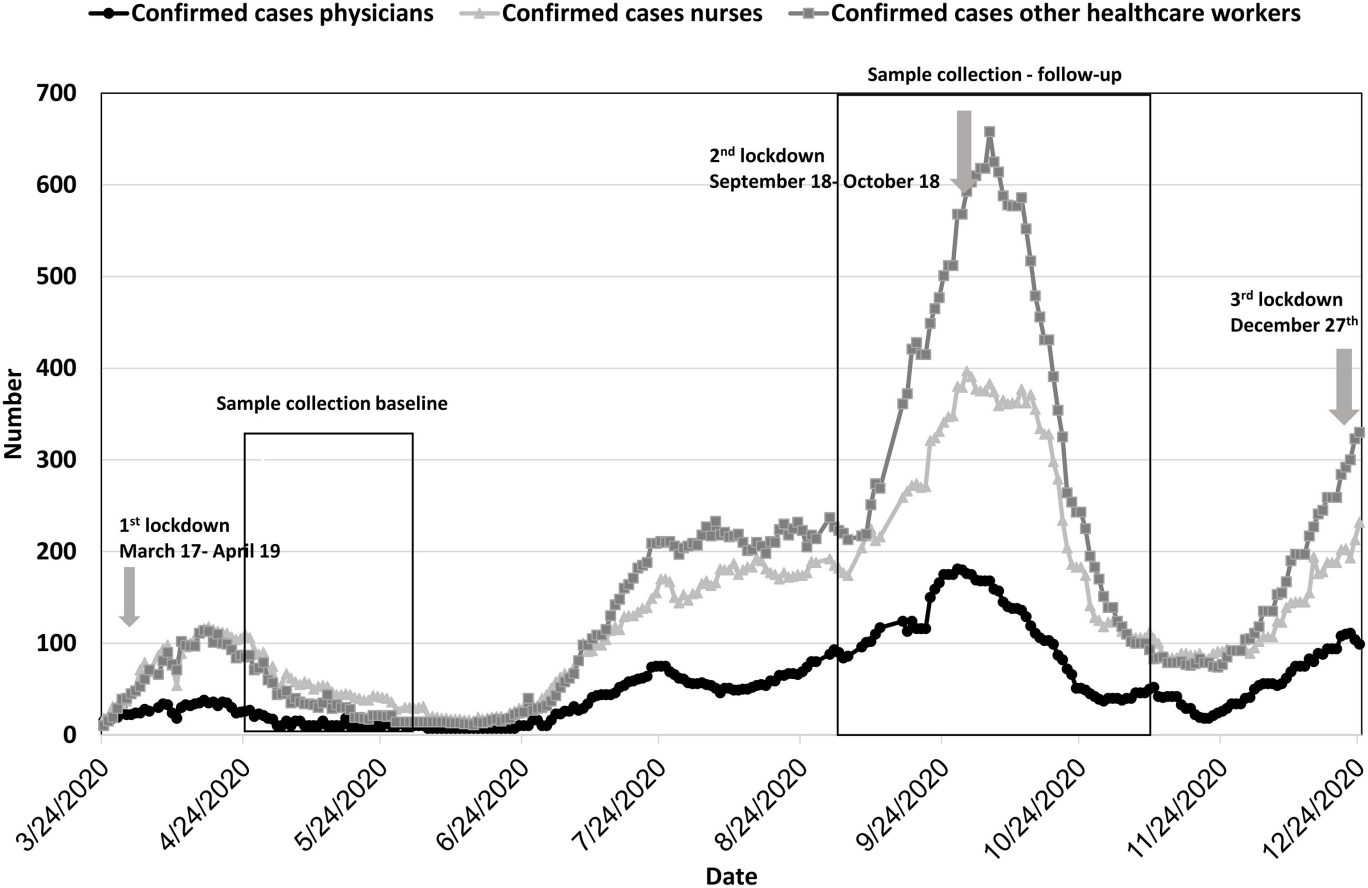
PCR-confirmed cases of SARS-CoV-2 in health care workers in general hospitals in Israel (*N* = 95,405). Black line—physicians; light gray line with triangles—nurses; dark gray line with squares—other.

The daily number of HCW employees of all general hospitals who had PCR-confirmed SARS-CoV-2 infection is presented in Figure 1. There were two peaks of SARS-CoV2 infection in HCWs, the first in mid-April 2020 and the second in mid-September 2020. Since December 2020, an increase in the number of cases has been observed. The incidence in HCWs corresponded to the incidence of COVID-19 in the general population in Israel.

### Sero-Epidemiological Studies

The participants' mean age was 39.6 years (SD 11.0), and 37.5% of them were males. The demographic and professional characteristics of participants in the follow-up assessment were comparable to that of the entire cohort (Table 1).

**Table 1:** Characteristics of the participants at baseline and follow-up assessments.

	**Baseline April–May 2020 *N* (%)**	**Follow-up September–November 2020 *N* (%)**
**Total**	874 (100.0%)	373 (100.0%)
**Medical center**		
Shamir (Assaf Harofe)	61 (7.0%)	–
Bilenson	166 (19.0%)	108 (29.0%)
HaSharon	41 (4.7%)	–
Hadassah Ein Kerem	66 (7.6%)	–
Wolfson	63 (7.2%)	30 (8.0%)
Laniado	68 (7.8%)	43 (11.5%)
Meir	66 (7.6%)	–
Shaare Zedek	121 (13.8%)	83 (22.3%)
Hillel Yaffe	222 (25.4%)	109 (29.2%)
**Sex**		
Males	328 (37.5%)	130 (34.9%)
Females	546 (62.5%)	243 (65.1%)
**Mean age (SD), years**	39.6 (11.0)	40.9 (11.3)
Missing	12 (1.3%)	5 (1.3%)
**Profession**		
Physician	341 (39.0%)	149 (39.9%)
Nurse	334 (38.2%)	128 (34.3%)
Other	188 (21.5%)	93 (24.9%)
Missing	11 (1.3%)	3 (0.8%)
**Years in the profession**		
0–3 years	253 (28.9%)	94 (25.2%)
4–10 years	196 (22.4%)	77 (20.6%)
>10 years	363 (41.5%)	180 (48.3%)
Missing	62 (7.1%)	22 (5.9%)

*SD, standard deviation*.

At baseline, 48.9% of the participants reported ever having worked in a coronavirus department vs. 55.2% at the follow-up assessment. Exposure to a confirmed COVID-19 patient outside the hospital was reported in 23.6% of the baseline participants and in 59.2% at the follow-up assessment. Performing a SARS-CoV-2 PCR test increased between baseline and follow-up, as well as the proportion of those who tested positive (Supplementary Table 1).

At baseline, 10 out of 874 participants tested positive for SARS-CoV-2 antibodies by both Roche and EUROIMMUN assays, yielding a prevalence of 1.1% (95% CI 0.6–2.1). Only three of these sero-positive participants had PCR-confirmed SARS-CoV-2 infection, one was asymptomatic and two had symptoms of fever, cough, fatigue, and sore throat.

At follow-up, 31 out of 372 HCW with usable samples tested positive for SARS-CoV-2 serum antibodies, yielding a sero-prevalence of 8.3% (95% CI 5.9–11.6). Among 369 participants with paired sera, four tested positive at both assessments, and 25 sero-converted; thus, the sero-incidence was estimated at 25/365 [6.9% (95% CI 4.7–9.9)].

Among 27 sero-positive participants who were tested by PCR in the past, 21 (77.8%) reported a positive result and reported symptoms of fever (*n* = 7), cough (*n* = 7), fatigue (*n* = 9), muscle pain (*n* = 9), and loss of taste/smell (*n* = 8).

### Factors Associated With the Prevalence of SARS-CoV-2 Serum Antibodies

A higher proportion of males was found in sero-positive than in sero-negative participants, as well as a higher proportion of nurses compared to physicians, but these differences were not statistically significant. The proportion of participants who reported ever working in a coronavirus department was higher in the sero-positive vs. sero-negative group (*P* = 0.056). A similar but not statistically significant (*P* = 0.098) trend was found for working in a coronavirus department in the past 3 month preceding the interview. No significant association was found between reports on exposure to a confirmed COVID-19 patient and SARS-CoV-2 sero-positivity (*P* = 0.166). However, the proportion of those who had been quarantined due to exposure to a confirmed COVID-19 case was higher among sero-positive vs. sero-negative personnel (*P* < 0.001). A similar result was found for having a family member who had been quarantined due to exposure to a confirmed COVID-19 patient (*P* = 0.003). Having a positive PCR result for the detection of SARS-CoV-2 was more common in the sero-positive vs. the sero-negative group (*P* < 0.001) (Table 2).

**Table 2:** Factors associated with the prevalence of SARS-CoV-2 serum antibodies at follow-up.

	**Positive for SARS-CoV-2 antibodies (*n* = 31)**	**Negative for SARS-CoV-2 antibodies (*n* = 341)**	***P*-value^a^**	**Unadjusted OR (95% CI)^b^**	***P*-value^b^**
**Sex**			0.101		0.105
Males	15 (48.4%)	115 (33.7%)		1.84 (0.88–3.86)	
Females	16 (51.6%)	226 (66.3%)		Reference	
**Mean age (SD), years**	40.7 (12.0)	40.9 (11.2)	0.900	0.99 (0.97–1.03)	0.893
**Profession**			0.246		0.100
Physician	10 (32.3%)	139 (41.1%)		Reference	
Nurse	15 (48.4%)	113 (33.4%)		1.88 (0.81–4.33)	0.141
Other	6 (19.4%)	86 (25.4%)		0.98 (0.35–2.81)	0.982
**Years in the profession**			0.561		0.553
0–3 years	10 (32.3%)	84 (26.3%)		Reference	
4–10 years	8 (25.8%)	69 (21.6%)		0.95 (0.35–2.54)	0.920
>10 years	13 (41.9%)	166 (52.0%)		0.64 (0.27–1.53)	0.316
**Ever worked in a coronavirus department**			0.056		0.061
Yes	22 (73.3%)	184 (55.3%)		2.23 (0.96–5.14)	
No	8 (26.7%)	149 (44.7%)		Reference	
**Worked in a coronavirus department in the past 3 months**			0.098		0.102
Yes	19 (63.3%)	170 (52.5%)		1.91 (0.88–4.13)	
No	11 (36.7%)	154 (47.5%)		Reference	
**Exposure to a confirmed COVID-19 patient in the past 3 months**			0.166		0.171
Yes	22 (73.3%)	199 (60.5%)		1.79 (0.78–4.16)	
No	8 (26.7%)	130 (39.5%)		Reference	
**Requested to be quarantined due to exposure to a confirmed COVID-19 patient**			<0.001		<0.001
Yes	16 (53.3%)	75 (22.7%)		3.90 (1.82–8.60)	
No	14 (46.7%)	256 (77.3%)		Reference	
**A quarantined family member due to exposure to a COVID-19 patient**			0.003		0.004
Yes	16 (51.6%)	87 (26.3%)		2.99 (1.42–6.31)	
No	15 (48.4%)	244 (73.7%)		Reference	
**SARS-CoV-2 test by PCR**			0.057		0.067
Yes	27 (87.1%)	237 (71.2%)		2.73 (0.93–8.02)	
No	4 (12.9%)	96 (28.8%)		Reference	
**Result of SARS-CoV-2 PCR test**			<0.001		<0.001
Positive	16 (61.5%)	4 (1.7%)		93.2 (26.29–330.33)	
Negative	10 (38.5%)	233 (98.3%)		Reference	

a *P-value was obtained by the chi-square test*;

b *P-value was obtained by bivariate logistic regression; OR, odds ratio; CI, confidence intervals. Some participants did not answer all questions, therefore totals might differ (Supplementary Table 2)*.

A multivariable model showed that males had higher odds to be sero-positive than females. Being asked to be in quarantine due to exposure to a confirmed COVID-19 patient was related to increased likelihood for SARS-CoV-2 sero-prevalence. A trend of a positive but non-statistically significant association was found between reporting a quarantine of a family member and SARS-CoV-2 antibody sero-positivity (*P* = 0.069). These models also showed that nurses were more likely than physicians to be sero-positive for SARS-CoV-2 antibodies (*P* = 0.076). The Nagelkerke *R* square (pseudo *R* square measure) for this model was 0.147.

Another model that included the same variables, in addition to SARS-CoV-2 PCR test result, showed a strong association between having a positive PCR result and SARS-CoV-2 sero-prevalence, and nurses had 4.26 higher odds to be sero-positive than physicians (*P* = 0.038). The Nagelkerke *R* square for this model was 0.555 (Table 3).

**Table 3:** Multivariable logistic regression model of factors associated with the prevalence of SARS-CoV-2 serum antibodies at follow-up.

	**Model 1^a^**		**Model 2^b^**	
	**Adjusted OR (95% CI)**	***P*-value**	**Adjusted OR (95% CI)**	***P*-value**
**Sex**
Males	2.52 (1.05–6.06)	0.039	4.77 (1.26–18.05)	0.021
Females	Reference			
**Profession**		0.164		0.087
Physician	Reference		Reference	
Nurse	2.38 (0.92–6.17)	0.076	4.26 (1.08–16.77)	0.038
Other	1.24 (0.37–4.16)	0.730	1.34 (0.22–7.99)	0.751
**Requested to be quarantined due to exposure to a confirmed COVID-19 patient**				
Yes	3.54 (1.58–7.89)	0.002	1.97 (0.59–6.59)	0.259
No	Reference		Reference	
**A quarantined family member due to exposure to a COVID-19 patient**				
Yes	2.09 (0.94–4.65)	0.069	2.17 (0.67–6.99)	0.196
No	Reference			
**Result of SARS-CoV-2 PCR test**				<0.001
Positive	Not included		109.5 (23.88–502.12)	
Negative			Reference	

a *P value = 0.672 by the Hosmer & Lemeshow test. Model summary Nagelkerke R square = 0.147*.

b *P value = 0.553 by the Hosmer & Lemeshow test. Model summary Nagelkerke R square = 0.555. OR, odds ratio; CI, confidence intervals*.

## Discussion

We showed a substantial increase in the risk of SARS-CoV-2 infection among HCWs between the first and second waves of COVID-19 in Israel. This was reflected in a 7-fold increase in the prevalence of SARS-CoV-2 serum antibodies between baseline and follow-up, from 1.1 to 8.3% during a 4–7 month period.

The estimated 1.1% prevalence of SARS-CoV-2 serum antibodies during the first wave of the epidemic is comparable to the reported estimates in HCWs in Germany, Greece, and Saudi Arabia (11, 12, 23). A massive increase in COVID-19 incidence occurred in Israel in parallel to the lifting of the first lockdown and especially with family vacations with crowding and the schools re-opening in September 2020. This change in the incidence in COVID-19 in the general population also affected HCWs, as shown in our study. These findings are particularly alarming, illustrating the risk of SARS-CoV-2 infection in vital workforces in times they are needed most, as during a second morbidity surge. This noteworthy finding reinforces the need to continually strengthen infection control and preventive measures among HCWs.

Despite the substantial increase in the prevalence of SARS-CoV-2 antibodies among HCWs in the second wave, the sero-prevalence remained relatively low (8.3%), being similar to a weighted sero-prevalence of 5.5% reported in a large sample of the general population during June–September (24). Our estimate is lower than the reported estimates of SARS-CoV-2 sero-prevalence in HCWs in the United Kingdom (14, 17), New York City (25), Spain (15), and Sweden (26, 27). Some of this dissimilarity might be the result of variation in study design and population, serological assays used in the study, and the circulation of SARS-CoV-2 in the community. Importantly, adherence to stringent infection control measures and high availability and utilization of personal protective equipment in medical facilities, as it is the case in Israel, seem to be critical in reducing the risk of SARS-CoV-2 infection in HCWs (11, 16). The increased incidence of seroconversion even with personal protective equipment might suggest that some of the new infections might have been acquired outside work. This also points to the need for continued vigilance and continuous quality management focusing on protection induced by adequate use of personal protective equipment to prevent transmission of the virus during patient–worker encounters.

We identified several risk factors for SARS-CoV-2 sero-prevalence. Interestingly, nurses compared to physicians were more likely to be sero-positive for SARS-CoV-2 antibodies. Common with our finding, others reported that nurses and nursing assistants were at increased risk for SARS-CoV-2 compared with physicians and other health professionals (27, 28). Under equitable and universal access to personal protective equipment, we assume that the increased risk for SARS-CoV-2 infection in nurses vs. physicians might be attributed to occupational characteristics, such as prolonged direct contact between nurses and patients, while such direct encounters between physicians and patients might be less frequent. A policy implemented during periods of increased volume of COVID-19 patients in hospitals is expanding the nurses' shifts up to 12 h, which might decrease the risk of exposure in the community and potential for cross-infection between HCWs. Hence, it is essential to assess the influence of such a policy on SARS-CoV-2 risk in HCWs. Collectively, our and others' findings (11, 15, 27, 28) reveal a differential risk for SARS-CoV-2 infection in various health professions, which should dictate locally tailored interventions to mitigate the risk of SARS-CoV-2 infection.

It has been shown that exposure to COVID-19 patients increases the risk for SARS-CoV-2 infection (27), especially if the contact occurred without using personal protective equipment (11, 16). In Israel, HCWs exposed to COVID-19 patients while using adequate personal equipment are exempted from quarantine, based on the protection afforded by use of this equipment; otherwise, the workers are requested to be quarantined for 10–14 days. This means that if exposure to a confirmed COVID-19 patient occurred outside the hospital, or while being at work, but not using personal protective equipment, HCWs were thus requested to be in quarantine. Indeed, we found no significant difference between HCWs who reported that they were exposed to COVID-19 patients and those who were not in terms of risk of SARS-CoV-2 sero-prevalence, likely since such exposure occurred while adequately using personal protective equipment. An intriguing finding is that HCWs who were requested to be in quarantine due to exposure to COVID-19 patients had a significant 3.5-fold higher likelihood to be sero-positive compared to those who were not in quarantine. Quarantine of HCWs is usually implemented if there is a tangible concern for SARS-CoV-2 infection that might be due to exposure to COVID-19 patients under improper use of personal protective equipment, or exposures that occurred in the community, which increase the risk for SARS-CoV-2 infection. Hence, our findings reinforce the so-far implemented policy of prevention and control of SARS-CoV-2 infections in HCWs in Israel, including quarantine when needed.

SARS-CoV-2-PCR positivity was reported by 7.6% of the participants at the follow-up assessment, which is lower than the identified 8.3% sero-prevalence. This finding supports the addition of sero-monitoring tools in the risk assessment of SARS-CoV-2 in HCWs and in other populations at risk especially when PCR testing is not performed systematically. Not surprisingly, having a positive PCR result for SARS-CoV-2 was strongly associated with sero-prevalence of SARS-CoV-2 antibodies. Previous sero-epidemiological studies mostly lacked information on SARS-CoV-2 PCR results. Hence, this finding supports previous reports on positive associations between the existence of prior symptoms consistent with COVID-19 and SARS-CoV-2 antibody sero-prevalence (13, 17, 25).

Our study has limitations. Mainly, we relied on a convenience sample of HCWs who were willing to take part in the study. We also focused on HCWs who work in hospitals that might not represent well HCWs in community clinics. Also, four of the nine hospitals elected not to participate in the follow-up assessment, and this resulted in a smaller sample size (*N* = 373) at the follow-up compared to baseline (*N* = 874). Nonetheless, the demographic and occupational profile of the participants at the follow-up assessment were similar to the baseline cohort, and we were able to identity several risk factors for SARS-CoV-2 antibody sero-positivity.

Our study has several strengths. First, the longitudinal design enabled the assessment of the change in sero-positivity between the first and second waves of COVID-19 in Israel. Second, it included HCWs from multiple centers, various regions, and tertiary and non-tertiary care hospitals. Third, it included HCWs who worked in dedicated coronavirus and non-coronavirus wards, as well as low-risk employees such as administrative staff. These elements increase the generalizability of our findings. Fourth, it provides a comprehensive assessment of potential exposures to SARS-CoV-2 infection that enabled the identification of risk factors with sufficient granularity to support policy-making. Fifth, in the classification of sero-positive individuals, we relied on a highly specific approach to reduce to the possibility of false positive findings.

In conclusion, in this longitudinal study of HCWs, we demonstrated a 7-fold increase in the sero-prevalence of SARS-CoV-2 antibodies among HCWs between the first and second waves of COVID-19 in Israel, which paralleled a substantial rise in the number of PCR-confirmed SARS-CoV-2 infections among HCWs across the country and in the general population. Despite this increase, the cumulative exposure to SARS-CoV-2 among HCWs in Israel remains relatively low, which reinforces the current rigorous policy of infection control, proper utilization of personal protective equipment, and quarantine. Interventions to reduce the risk of SARSCoV-2 in HCWs should prioritize nurses and focus on decreasing direct unprotected contact with SARS-CoV-2 patients that leads to exposure and a need for quarantine. These measures should be continued in parallel to the roll-out of COVID-19 vaccines in HCWs and the general population.

## Data Availability Statement

The datasets presented in this article are not readily available because legal restrictions apply. Requests to access the datasets should be directed to kmuhsen@tauex.tau.ac.il.

## Ethics Statement

The study protocol was approved by Institutional Review Boards of participating hospitals and by the Ethics Committee of Tel Aviv University. All participants volunteered for the survey and consented in writing.

## Author Contributions

KM, JB, AA, YC, DC, and EK conceived and designed the study. MS, JB, EK, and WN made significant contributions in the coordination of the study. MS, JB, AA, EK, WN, JM, RC, PS, DM, SB, DB-D, BR, TG, AN, YW-W, and YM performed the studies, including enrollment and sample and data collection. AB, YZ, NH, and OA-C performed the laboratory experiments. SG, DC, and KM analyzed the data. KM and DC drafted the manuscript. All authors critically revised the manuscript for important intellectual content, gave final approval for the version to be published, and agreed to be accountable for all aspects of the work in ensuring that questions related to the accuracy or integrity of any part of the work are appropriately investigated and resolved.

## Conflict of Interest

The authors declare that the research was conducted in the absence of any commercial or financial relationships that could be construed as a potential conflict of interest.
