# Supplementary material for: Sero-Prevalence and Sero-Incidence of Antibodies to SARS-CoV-2 in Health Care Workers in Israel, Prior to Mass COVID-19 Vaccination
Source: Front Med (Lausanne). 2021 Jun 24;8:689994. doi: 10.3389/fmed.2021.689994 (PMC8268152; doi:10.3389/fmed.2021.689994)
Supplement: Supplementary file 1 [file Data_Sheet_1.pdf]

Supplementary Table 1: Exposures to SARS-CoV-2 among the participants at baseline and follow-up assessments

	Baseline April-May 2020	Follow-up September-November 2020
	Number (%)	Number (%)
**Total**	874 (100.0%)	373 (100.0%)
**Ever worked in a coronavirus department**		
Yes	340 (48.9%)	206 (55.2%)
No	486 (55.6%)	158 (42.4%)
Missing	48 (5.5%)	9 (2.4%)
**Exposure to a confirmed COVID-19 patient**		
Yes	206 (23.6%)	221 (59.2%)
No	586 (67.0%)	139 (37.3%)
I do not know	55 (6.3%)	-
Missing	27 (3.1%)	13 (3.5%)
**A quarantined family member due to exposure to a COVID-19 patient**		
Yes	81 (9.3%)	103 (27.6%)
No	765 (87.5%)	260 (69.7%)
I do not know	6 (0.7%)	0 (0%)
Missing	22 (2.5%)	10 (2.7%)
**Have you been tested for SARS-CoV-2 (PCR nasopharyngeal swab)?** [a]		
Yes	310 (35.5%)	265 (71.0%)
No	540 (61.8%)	100 (26.8%)
Missing	24 (2.7%)	8 (2.1%)
**Result of SARS-CoV-2 PCR test among those tested** [ab]		
Positive	5 (1.6%)	20 (7.6%)
Negative	299 (96.5%)	244 (92.1%)
Missing	6 (1.9%)	1 (0.3%)

[a] At the follow-up assessment the question was regarding a PCR test for SARS-CoV-2 in the past 3 months (i.e. not including the PCR test at the baseline assessment). [b] The positive PCR results for SARS-CoV-2 were taken during September-October, i.e. not included the positives at baseline assessment

Supplementary table 2: Missing information by variable

Variable	Number (%) with missing information
age	5 (1.3%)
Profession	3 (0.8%)
Years in the profession	22 (5.9%)
Ever worked in a coronavirus department	9 (2.4%)
Worked in a coronavirus department in the past 3 months	18 (4.8%)
Exposure to a confirmed COVID-19 patient in the past 3 months	13 (3.5%)
Requested to be quarantined due to exposure to a confirmed COVID-19 patient	11 (3.0%)
A quarantined family member due to exposure to a COVID-19 patient	10 (2.7%)
SARS-CoV-2 test by PCR	8 (2.2%)
Result of SARS-CoV-2 PCR test	1 (0.4%)

Percent out of 372 participants with useable serum samples
